# Mechanisms and therapeutic prospects of hypoxia-inducible factor 1-alpha in acute kidney injury: a systematic review

**DOI:** 10.3389/fcell.2025.1660433

**Published:** 2026-01-12

**Authors:** Peng Kang, Fan Cheng

**Affiliations:** Department of Urology, Renmin Hospital of Wuhan University, Wuhan, China

**Keywords:** acute kidney injury, hypoxia-inducible factor-1α, ischemia-reperfusion injury, mitochondrial dysfunction, oxidative stress

## Abstract

**Background:**

Acute Kidney Injury (AKI) poses a significant global health challenge, with increasing incidence and mortality rates, and profoundly impacts long-term outcomes, including progression to chronic kidney disease. Ischemia-reperfusion injury (IRI) is a major cause of AKI, in which hypoxia-inducible factor-1α (HIF-1α) plays a complex and dual role.

**Methods:**

This review systematically analyzes the regulatory functions of HIF-1α in renal IRI, focusing on molecular mechanisms involving oxidative stress, apoptosis, inflammation, and tissue repair.

**Results:**

Emerging evidence from preclinical studies demonstrates that HIF-1α orchestrates key adaptive responses in renal IRI, including the regulation of mitophagy, management of endoplasmic reticulum stress, and induction of metabolic reprogramming toward glycolysis.

**Conclusion:**

Targeting HIF-1α represents a promising therapeutic strategy for AKI. Advances in HIF-1α-modulating therapies, particularly HIF prolyl hydroxylase inhibitors, offer novel avenues for both prevention and treatment. These findings underscore the potential for HIF-1α-centered therapies to mitigate AKI progression and improve clinical outcomes.

## 1 Introduction

AKI, characterized by a rapid decline in glomerular filtration rate (GFR) and nitrogenous waste accumulation, has increased significantly globally over the past two decades (Folkestad et al., 2020). Its incidence is approximately 10%–15% among hospitalized patients and exceeds 50% in intensive care unit patients and is associated with a fourfold increase in mortality risk (White et al., 2023). Renal IRI, a key pathological mechanism often secondary to sepsis, trauma, or major surgery, involves both direct ischemic damage and reperfusion-induced inflammatory cascades (Cao et al., 2021; Sha et al., 2022). It is also closely associated with microvascular dysfunction (Pang et al., 2018; Pang et al., 2017) and reduced renal plasma flow (RPF) (Huang et al., 2016). Given its high morbidity and mortality rates (Wang et al., 2020), understanding the molecular basis of IRI is crucial for early AKI detection and prevention.

The kidneys, being highly perfused organs, are especially vulnerable to ischemia-induced injury, rapidly developing oxidative stress due to ROS, along with inflammation and apoptosis, particularly in the proximal tubules (Hong et al., 2017; Zhou et al., 2022; Huang J. et al., 2022). Reperfusion further exacerbates injury; ROS levels surge early and persist for 24 h (Jia et al., 2019), reducing nitric oxide (NO) bioavailability and worsening vasoconstriction (Ran et al., 2022). ROS accumulation plays a central role in both the ischemic and reperfusion phases. Under normal physiological conditions, the production of ROS is balanced by antioxidant systems, including enzymes such as superoxide dismutase (SOD) (Prieux-Klotz et al., 2022; Rai et al., 2018), but hypoxia disrupts mitochondrial metabolism and accelerates ROS generation (Vestergaard et al., 2020). HIF-1α exhibits a dual regulatory role: it contributes to a damaging ROS-HIF-1α feedback loop (Dai et al., 2019), yet also suppresses toxic ROS (Chun et al., 2010). Its specific regulatory network in renal IRI requires systematic elucidation. This review focuses on the key mechanisms by which HIF-1α influences renal IRI.

## 2 Pathophysiological functions of HIF-1α

HIF is a heterodimeric transcription factor composed of α (HIF-1α, HIF-2α) and β subunits (Li et al., 2017). The stability of the HIF-1α subunit is a key determinant of HIF activity. Under normoxic conditions, prolyl hydroxylase domain enzymes (PHDs) hydroxylate HIF-1α, promoting its recognition by the von Hippel-Lindau (pVHL) E3 ubiquitin ligase complex, which targets it for proteasomal degradation. In contrast, under hypoxic conditions, hydroxylation is inhibited, enabling HIF-1α accumulation, nuclear translocation, dimerization with HIF-1β, and activation of target genes involved in angiogenesis (Semenza, 2003), apoptosis (Carmeliet et al., 1998), and metabolism (Denko, 2008). The cellular response to hypoxia is the core mechanism for inducing HIF-1α expression and activating the HIF-1 complex (Bryant et al., 2018). During hypoxia, pVHL loses its ability to recognize HIF-1α, leading to its accumulation and translocation to the cell nucleus, where it forms a functional dimer with HIF-1β (Colbert et al., 2015). Therefore, the transcriptional activity of HIF-1 fundamentally depends on the protein stability of HIF-1α (He et al., 2015).

In conditions of inadequate perfusion, hypoxia-induced accumulation of HIF-1α activates transcription of key glycolytic genes, accelerating ATP production to maintain cellular energy homeostasis. Notably, HIF-1β, as a structural subunit, is constitutively expressed and not regulated by oxygen concentration (Giaccia et al., 2003). Under physiological conditions, HIF-1 plays a role in normal processes such as embryonic development. However, under pathological conditions, abnormal accumulation of HIF-1 is closely associated with the development of ischemic diseases, tumor formation, and infectious diseases (Chen et al., 2015). Further studies have revealed that HIF-1α activation stimulates the expression of pyruvate kinase M2 (PKM2), which in turn, enhances HIF-1α transcriptional activity through a positive feedback loop, forming a self-amplifying circuit (Zhang et al., 2021). During tissue ischemia-reperfusion, β-catenin binds to HIF-1α and significantly enhances its stability under hypoxic conditions, potentially exacerbating tissue damage during the reperfusion. The stability of HIF-1α is finely regulated by its oxygen-dependent degradation domain (ODDD), which contains the catalytic site of PHDs (Wei et al., 2023). PHDs use oxygen molecules as substrates to hydroxylate specific proline residues on HIF-1α, forming a key biochemical switch linking oxygen concentration changes to the HIF signaling pathway (Hu et al., 2020). In addition to the classical oxygen-dependent degradation pathway, recent studies have shown that RNA-binding motif protein 4 (RBM4) can enhance HIF-1α expression by promoting its mRNA translation through an oxygen-independent mechanism (Chang and Lin, 2019).

**FIGURE 1:**
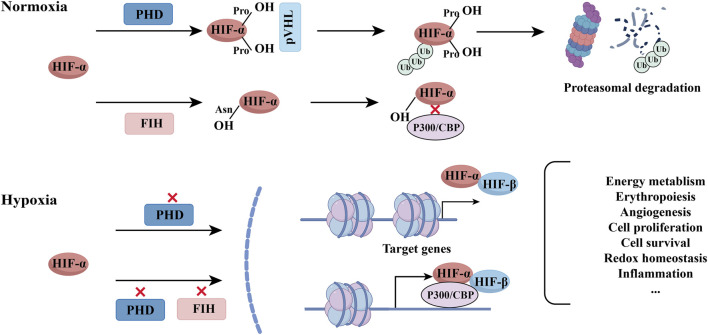
Regulation and transcriptional activation of HIF-1α under normoxic and hypoxic conditions. Under normoxia, prolyl hydroxylase domain (PHD) enzymes hydroxylate HIF-1α, leading to its recognition by the von Hippel–Lindau (pVHL) E3 ubiquitin ligase complex and subsequent proteasomal degradation. Concurrently, factor inhibiting HIF-1 (FIH) hydroxylates an asparagine residue in the C-terminal transactivation domain (C-TAD) of HIF-1α, preventing its interaction with transcriptional coactivators (e.g., p300/CBP). Under hypoxia, the inhibition of PHD and FIH activities allows for HIF-1α stabilization and full transcriptional competence. Stabilized HIF-1α translocates to the nucleus, dimerizes with HIF-1β, and the heterodimer binds to hypoxia-response elements (HREs) in the DNA. The recruitment of coactivators (e.g., p300/CBP) to the HIF-1 complex then initiates the transcription of target genes involved in angiogenesis, metabolism, and cell survival.

On a metabolic level, HIF-1α promotes anaerobic glycolysis and regulates mitochondrial quality control. Masoud et al. (2015) demonstrated that HIF-1α enhances mitophagy, maintaining energy homeostasis in AKI (Masoud and Li, 2015). In the context of inflammation, tubular-derived extracellular vesicles (EVs) stabilize macrophage HIF-1α, inducing glycolytic reprogramming and promoting IL-1β, TNF-α, and TGF-β1 release, thereby exacerbating interstitial inflammation and fibrosis (Chen et al., 2019) (Figure 1).

## 3 HIF-1α and renal ischemia: novel evidence for dual roles

### 3.1 Regulation and functional diversity in AKI

The dynamic expression of HIF-1α critically influences the outcomes of AKI. In mouse models of IRI, sustained HIF-1α activation is associated with severe AKI and fibrosis, whereas administration of the HIF-1α inhibitor PX-478 ameliorates the transition from AKI to chronic kidney disease (CKD) (Li et al., 2021a). In a rat model of lipopolysaccharide (LPS)-induced AKI, both HIF-1α and aquaporin-1 (AQP1) were significantly upregulated within 12 h, concomitant with hypoglycemia, enhanced glycolysis, and a pro-inflammatory shift (elevated IL-6/TNF-α and reduced IL-10). Inhibition of HIF-1α reversed these effects, and knockdown of AQP1 attenuated HIF-1α upregulation (Diao et al., 2024), indicating a finely tuned regulatory interplay linked to disease progression.

In ischemic AKI, mitophagy activation is characterized by upregulation of microtubule-associated protein 1 light chain 3, PTEN-induced kinase 1, and PARKIN, along with a decrease in p62. HIF-1α binds to the promoter of miR-140-5p, leading to its downregulation and subsequent promotion of mitophagy through the PARKIN pathway (Zhang Q. et al., 2022). In contrast-induced AKI (CI-AKI), genetic ablation of NLRP3 or caspase-1 enhances hypoxia signaling and mitophagy. Furthermore, the HIF prolyl hydroxylase inhibitor Roxadustat stabilizes HIF-1α, which activates Bcl-2/adenovirus E1B 19 kDa interacting protein 3 (BNIP3)-mediated mitophagy and confers tubular protection (Lin et al., 2021).

### 3.2 The association between HIF-1α and AKI prognosis

Clinical studies in AKI patients have shown that renal Klotho protein levels are significantly negatively correlated with AKI severity, and its low expression is associated with a poor short-term prognosis. Growth Differentiation Factor-15 (GDF15), a stress-responsive cytokine produced by various cell types in response to cellular stress, tissue injury, hypoxia, and oncogene activation, has been shown to protect Klotho expression. In transcriptomic analyses of experimental acute kidney injury and renal fibrosis, GDF15 was the most significantly upregulated gene in the GDF family. Consistently, *Gdf15*-deficient mice exhibit more severe AKI, while GDF15 overexpression or exogenous administration protects the kidneys and maintains Klotho expression (Valino-Rivas et al., 2022; Seo et al., 2015).

In a rat model of rhabdomyolysis-associated acute kidney injury (RM-AKI), after glycerol injection, Bach1 mRNA levels sharply increased at 3 h and remained elevated for 12 h. Conversely, its nuclear protein levels significantly decreased after 3 h, and cytoplasmic protein levels markedly increased after 6 h. Simultaneously, mRNA and protein expression of heme oxygenase-1 (HO-1) were significantly upregulated, suggesting that dynamic changes in Bach1 expression may mediate heme metabolism and potential renal protective mechanisms during AKI (Yamaoka et al., 2017). These stress-responsive pathways, including the upregulation of GDF15 and HO-1, may intersect with HIF-1α signaling, reflecting a coordinated hypoxia-adaptive response during AKI.

In patients with ANCA-associated vasculitis with glomerulonephritis (ANCA-GN), the levels of kidney injury molecule-1 (KIM-1) in the blood were elevated at diagnosis, decreased after induction therapy, and were associated with the severity of kidney injury and the need for renal replacement therapy; Notably, unlike other pro-inflammatory molecules, KIM-1 is primarily associated with acute tubular necrosis and interstitial fibrosis/tubular atrophy (IF/TA), while showing weaker associations with interstitial infiltration or glomerular involvement, suggesting that KIM-1 may serve as a potential biomarker for acute kidney injury and tubulointerstitial damage in ANCA-GN (Brilland et al., 2023). Research indicates that KIM-1 enhances HIF-1α activation thereby exacerbating renal tubulointerstitial inflammation (Chen et al., 2023; Kamel et al., 2019). This mechanism becomes particularly pronounced in ischemia-reperfusion injury, highlighting HIF-1α′s pivotal regulatory role in kidney damage.

Sustained HIF-1α activation promotes fibrosis; PX-478 given from day five post-IRI attenuated AKI-to-CKD progression (Li et al., 2021a). In sepsis-associated AKI (SA-AKI), elevated urinary miR-340-5p distinguished SA-AKI patients and correlated with poor outcomes, potentially by targeting KDM4C to regulate tubular injury (Pu et al., 2025). Low serum bicarbonate (HCO_3_^−^) levels predicted poor renal outcomes at 6 months (AUC = 0.798); when combined with serum creatinine (Scr), the predictive accuracy improved (AUC = 0.952) (Che et al., 2014). The urinary angiotensinogen-to-creatinine ratio was higher in patients with KDIGO stage 3 AKI and correlated with Scr levels at 3 months (Akin et al., 2023), suggesting that urinary angiotensinogen levels may serve as an indicator of AKI severity, and monitoring its dynamic changes could aid in prognostic assessment and patient management.

### 3.3 The dual mechanism of HIF-1α during tissue ischemia

Ischemia induces a hypoxic metabolic state, leading to ATP depletion and acidosis. In response, HIF-1α protein stabilizes, translocates to the nucleus, forms a complex with HIF-1β, and transactivates protective genes such as vascular endothelial growth factor (VEGF). Studies employing conditional knockout mice have been pivotal in elucidating the cell-specific functions of HIF-1α in AKI. For instance, tubule-specific deletion of HIF-1α (HIF‐1α^flox/flox^: cadherin16-Cre^+^ mice) exacerbates renal injury, increasing tubular apoptosis, ROS production, and functional impairment following ischemic insult (Fu et al., 2020). Similarly, proximal tubule-specific knockout of HIF-1α aggravates tubular injury and mitochondrial dysfunction in diabetic nephropathy models (Jiang N. et al., 2020). Conversely, the role of HIF-1α in fibrosis is complex and phase-dependent. Genetic ablation of its transactivation domains significantly ameliorates tubulointerstitial fibrosis in chronic hypoxia-induced CKD models (Zhang et al., 2025), highlighting a potential pro-fibrotic role in persistent injury. Tubular HIF-1α knockout also ameliorates fibrosis mediated by proteins like LIM and senescent cell antigen-like domains 1 (Han et al., 2024). Its role in ferroptosis is multifaceted, as proximal tubule-specific knockout (HIF‐1α^flox/flox^ Ggt1‐Cre mice) intensifies ferroptotic damage in some models (Kang et al., 2025). These *in vivo* findings robustly support a protective role for tubular HIF-1α in acute injury phases by attenuating apoptosis, oxidative stress, and mitochondrial damage, consistent with studies showing that HIF-1α overexpression reduces caspase-3/9/Bax, increases Bcl-2, lowers pro-inflammatory cytokines (IL-6/IL-1β/TNF-α), and elevates anti-inflammatory IL-10 (He et al., 2021; Wang et al., 2022; Liu et al., 2023).

PTEN exerts protection via the PI3K/Akt/HIF-1α/mTOR pathway by inhibiting excessive Akt phosphorylation and promoting protective autophagy (Wang et al., 2021). MUC1 enhances HIF-1α activity; Muc1^−/−^ mice showed impaired HIF-1 target gene induction (glycolysis), prolonged AMPK activation, metabolic stress, and increased AKI susceptibility (Pastor-Soler et al., 2015).

Mild ischemia during tumor resection causes minimal HIF-1α reduction without functional impact (Mikkelsen et al., 2021). Injury severity is key: mild injury increases NRF2 nuclear localization aiding recovery, while severe injury suppresses NRF2, worsening fibrosis (Bondi et al., 2022). Elevation of HIF-1α enhances tubular mitophagy via activation of BNIP3 and related pathways, thereby promoting mitochondrial quality control and improving ischemic tolerance (Li Z.-L. et al., 2023). However, prolonged hypoxia triggers irreversible damage; HIF-1α-dependent exosomal miR-23a from injured tubules activates macrophages, promoting tubulointerstitial inflammation (Li et al., 2019).

**FIGURE 2:**
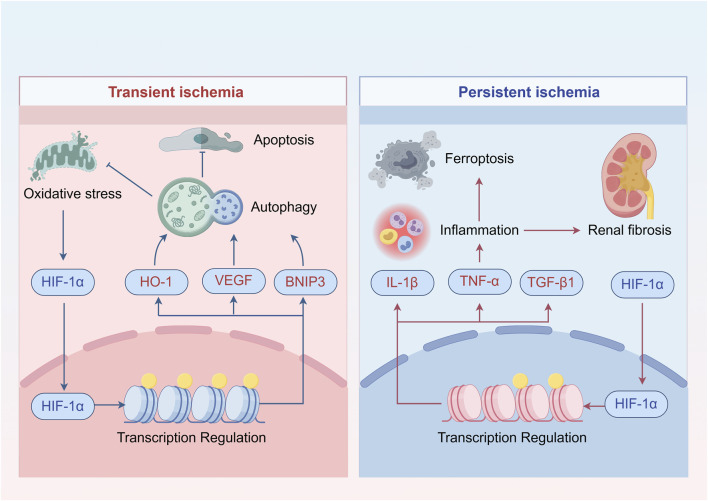
The dual role of HIF-1α in renal ischemia. The mechanism of HIF-1α is context-dependent. (Left Panel) Adaptive Response in Transient Ischemia: During brief ischemic periods, hypoxia and oxidative stress stabilize HIF-1α. Nuclear HIF-1α transactivates target genes such as HO-1, VEGF, and BNIP3, promoting cytoprotective processes including autophagy, mitigation of oxidative stress and apoptosis, thereby enhancing ischemic tolerance. (Right Panel) Maladaptive Response in Prolonged Hypoxia: Under sustained hypoxic stress, persistent HIF-1α activation drives the expression of pro-inflammatory and pro-fibrotic mediators (e.g., IL-1β, TNF-α, TGF-β1). This promotes tubulointerstitial inflammation, ferroptosis, and ultimately contributes to renal fibrosis, exacerbating tissue damage.

In conclusion, HIF-1α acts as a “double-edged sword” during ischemia: it is protective in transient ischemia by inhibiting apoptosis, but detrimental during prolonged and irreversible injury by promoting inflammation (Figure 2).

## 4 HIF-1α and renal ischemia-reperfusion: novel perspectives on protection

### 4.1 Core characteristics of renal ischaemia-reperfusion injury

Renal IRI is characterized by an early ROS burst (peaking at 24 h) followed by a pro-inflammatory cytokine cascade (IL-1β, TNF-α, IL-6) (Jia et al., 2019; Rossi et al., 2018). These events lead to endothelial dysfunction, microvascular injury, reduced GFR and renal blood flow (RBF), parenchymal damage, and persistent dysfunction. Oxidative stress is a key mechanism underlying IRI, primarily through ROS-induced cellular damage and death (Ishii et al., 2024). Additionally, the complement system fragments C3a and C5a play important roles by promoting inflammatory cell infiltration and the release of inflammatory mediators, thereby exacerbating renal injury (Peng et al., 2012). At the cellular level, IRI-induced apoptosis and necrosis are central pathological features. Studies have revealed that regulated necrosis, including necroptosis and ferroptosis, contributes significantly to renal IRI. These processes lead to the release of cellular contents through membrane rupture, triggering inflammatory responses and immune system activation (Pefanis et al., 2019). Protein kinase B1 (Akt1) also plays a key role in renal tubular cell apoptosis and inflammatory responses, and its deficiency exacerbates renal injury (Kim et al., 2020). At the molecular level, renal IRI involves the activation of multiple signaling pathways. The nuclear factor κB (NF-κB) signaling pathway is particularly important in mediating inflammatory responses, and its inhibition can alleviate inflammation and damage caused by IRI (Zou et al., 2021). Furthermore, phosphodiesterase inhibitors have demonstrated potential therapeutic effects in renal IRI by improving renal function through the regulation of intracellular signaling pathways (Thapa et al., 2021).

### 4.2 HIF-1α-mediated vascular repair mechanism

**FIGURE 3:**
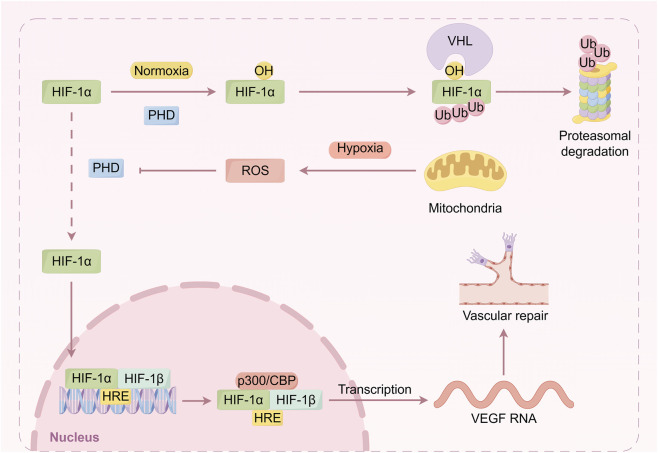
HIF-1α-mediated mechanisms of vascular repair and cytoprotection in renal ischemia-reperfusion injury. This diagram illustrates the central role of HIF-1α in coordinating the response to hypoxic stress. Under normoxia, HIF-1α is hydroxylated by prolyl hydroxylase domain enzymes (PHDs), recognized by the pVHL E3 ubiquitin ligase complex, and targeted for proteasomal degradation. Under hypoxia, PHD activity is inhibited (e.g., by mitochondrial ROS), leading to HIF-1α stabilization. Stabilized HIF-1α translocates to the nucleus, dimerizes with HIF-1β, and the complex binds to hypoxia-response elements (HREs). The subsequent recruitment of coactivators (e.g., p300/CBP) transactivates target genes. A key mediator is vascular endothelial growth factor (VEGF), a master regulator of angiogenesis, whose upregulation promotes vascular repair.

HIF-1α plays a crucial role in vascular repair. Endothelial HIF-1α promotes repair and resolution of lung injury via Forkhead box M1 (FoxM1) (Huang et al., 2019; Evans et al., 2022). Tubular HIF-1α promotes angiogenesis both directly, by upregulating VEGF (Xue et al., 2022), and indirectly, through miR-21–mediated suppression of TSP-1 (Xu et al., 2017). Additionally, HIF-1α enhances nitric oxide (NO) production by upregulating nitric oxide synthase (NOS) expression, thereby improving hemodynamics and further promoting vascular repair (Han et al., 2021). Beyond angiogenesis, HIF-1α mitigates kidney damage by modulating inflammatory responses. It downregulates the expression of pro-inflammatory cytokines and inhibits the infiltration of inflammatory cells, thereby reducing inflammation-induced damage (Li B. Y. et al., 2020). HIF-1α also confers protection by regulating specific microRNAs; for instance, it upregulates miR-21 to inhibit apoptosis and promote cell survival (Jia et al., 2017). Furthermore, HIF-1α protects renal tubular cells from ischemia-reperfusion injury by regulating mitochondrial autophagy. It promotes BNIP3-mediated mitophagy to clear damaged mitochondria, reduce reactive oxygen species production, and subsequently mitigate cell apoptosis and tissue damage (Fu et al., 2020). In kidney transplantation models, the expression level of HIF-1α in the donor kidney directly determines its capacity to adapt to hypoxic stress and repair damage, ultimately influencing transplantation outcomes (Duan and Qin, 2019). In summary, HIF-1α facilitates vascular repair in renal ischemia-reperfusion injury through multiple pathways, including promotion of angiogenesis, regulation of inflammatory responses, and protection of mitochondrial function. These mechanisms collectively mitigate renal injury, promote tissue repair, and identify potential therapeutic targets (Figure 3).

### 4.3 Multidimensional expansion of cell protection mechanisms

HIF-1α regulates necroptosis via the miR-26a/TRPC6 axis in macrophages and pulmonary arterial smooth muscle cells, thereby influencing oxidative stress and inflammation (Ma et al., 2023; Mal et al., 2024). Besides, Necrostatin-1 attenuated renal ischemia-reperfusion injury through mediation of the HIF-1α/miR-26a/TRPC6/PARP1 signaling pathway (Shen et al., 2019). Mechanistic studies demonstrated that HIF-1α directly binds to the promoter region of miR-26a, and that TRPC6 is a potential target gene of miR-26a (Shen et al., 2019). Lentivirus-mediated HIF-1α overexpression improved renal function while reducing inflammation and apoptosis in rats (Li X. et al., 2020). HIF-1α expression is critical for determining the adaptability of a transplanted kidney to hypoxic stress, and also contributes to damage repair, ultimately influencing transplant outcomes (Han et al., 2019). In renal tubules, elevated HIF-1α enhances the antioxidant capacity of epithelial cells. It stimulates adenosine production and upregulates CD39 and CD73 expression to prevent further renal injury (Zhou et al., 2018). Therefore, during renal ischemia-reperfusion, extensive tubular apoptosis due to hypoxia, combined with severe reperfusion-induced oxidative stress, exacerbates tissue damage. Insufficient HIF-1α expression is a key pathogenic factor that leads to sustained injury mediated by inflammation and oxidative stress from tubular epithelial cells, while repair mechanisms are impaired due to inadequate HIF-1α levels. HIF-1α enhances the ischemia tolerance of renal tubular epithelial cells through BNIP3-mediated mitophagy. The surge of oxidative stress during reperfusion disrupts endoplasmic reticulum (ER) function, resulting in overactivation of the unfolded protein response and induction of apoptosis. The HIF-1α/BNIP3 axis protects the kidney from ischemia-reperfusion injury (IRI) by promoting ER autophagy (reticulophagy) (Zhao et al., 2025). Thus, inadequate HIF-1α expression is a major contributor to tissue damage during reperfusion, and elevating HIF-1α levels may promote nephron survival and better functional preservation.

The protective role of HIF-1α is injury-severity dependent: mild ischemia promotes NRF2 nuclear translocation and enhances antioxidant responses, whereas severe ischemia suppresses NRF2 and exacerbates damage (Jia et al., 2022). Prolonged ischemia triggers the release of exosomes from irreversibly injured tubules, which stabilize HIF-1α in macrophages, induce glycolysis, and promote the release of IL-1β, TNF-α, and TGF-β1, thereby worsening inflammation and fibrosis (Jia et al., 2022). Persistent glycolysis may lead to acidosis and lactate accumulation. In conclusion, precise regulation of HIF-1α is essential for nephron protection during reperfusion injury.

## 5 Pathological mechanisms of HIF-1α in AKI

### 5.1 HIF-1α and ferroptosis in acute kidney injury

**FIGURE 4:**
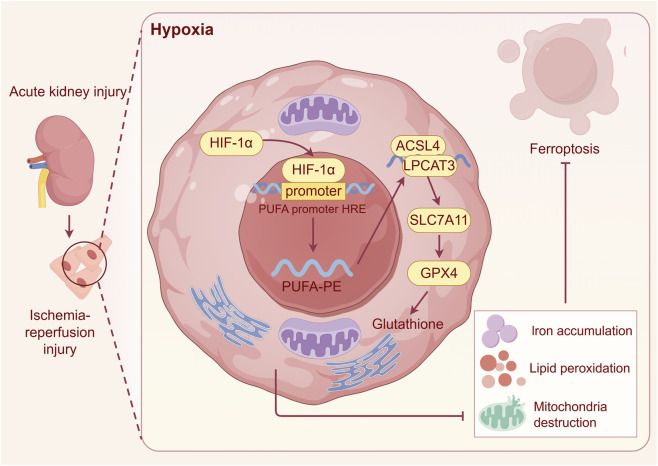
Diagram summarizing the protective role of HIF-1α against ferroptotic injury. Under hypoxic or ischemic conditions, HIF-1α activation upregulates antioxidant and metabolic targets that collectively reduce iron accumulation, suppress lipid peroxidation, and preserve mitochondrial integrity. Specifically, HIF-1α enhances the expression of SLC7A11 and GPX4, maintaining glutathione-dependent detoxification of lipid peroxides, while limiting ACSL4-mediated incorporation of polyunsaturated fatty acids (PUFAs) into membrane phospholipids (PUFA-PE). By modulating the PUFA-PE metabolism and the ACSL4–SLC7A11–GPX4 regulatory axis, HIF-1α attenuates ferroptosis-associated mitochondrial injury and oxidative stress.

Ferroptosis, an iron-dependent form of cell death driven by lipid peroxidation, plays a critical role in the progression of acute kidney injury. Mitochondria, central organelles in iron metabolism and homeostasis, contain mitochondrial ferritin, which serves to sequester and store iron in a non-reactive form, thereby mitigating iron-catalyzed oxidative damage within the organelle (Wu H. et al., 2021). However, mitochondrial iron accumulation promotes the generation of mitochondrial ROS, ultimately leading to ferroptosis. Typical features of ferroptosis include the inhibition of glutathione peroxide 4 (GPX4) activity and the excessive accumulation of lipid ROS. Although direct studies linking HIF-1α to ferroptosis are limited, several lines of indirect evidence suggest a potential protective role. As a key regulator of cellular homeostasis, HIF-1α is known to confer protection in RIR injury. Whether HIF-1α activation alleviates renal injury by resisting the ferroptosis process in AKI requires further investigation. The PHD inhibitor FG-4592 stabilizes HIF-1α, upregulates SOD2 expression, and reduces lipid peroxidation in CI-AKI (Wu M. et al., 2021). Mechanistic studies revealed that FG-4592 significantly decreased malondialdehyde levels in renal tubular epithelial cells while increasing GPX4 expression. In animal models, FG-4592 pretreatment markedly reduced the expression of ferroptosis markers in kidneys after ischemia-reperfusion, and this protective effect was abolished in renal tubule-specific HIF-1α knockout mice, confirming the necessity of HIF-1α in this process (Yang et al., 2023). Additionally, HIF-1α may regulate glutathione synthesis by modulating the cystine/glutamate antiporter system, thereby maintaining cellular antioxidant capacity. Under hypoxic conditions, HIF-1α upregulates hepcidin expression, inhibiting intestinal iron absorption and macrophage iron release, which reduces free iron accumulation. Notably, the anti-androgen drug bicalutamide selectively damages renal mesangial cells, inducing mitochondrial dysfunction through mechanisms such as enhanced lactate dehydrogenase release and promotion of ROS production. Bicalutamide regulated the HIF-1 pathway via ROS-induced damage, increasing cellular susceptibility to ferroptosis and ultimately contributing to renal injury (Chen et al., 2020) (Figure 4).

### 5.2 Regulation of oxygen metabolism in acute kidney injury by HIF-1α

HIF-1α plays a crucial role in regulating oxygen metabolism in AKI. In a LPS-induced AKI rat model, HIF-1α protein levels were significantly upregulated within 12 h, accompanied by reduced blood glucose concentrations and enhanced glycolytic activity, suggesting that HIF-1α facilitates metabolic adaption tohypoxia by regulating glucose metabolism (Diao et al., 2024). Administration of HIF-1α inhibitors reversed these effects, further supporting its critical role in glycolytic regulation. In a human model of hypoxia/reoxygenation (H/R) injury renal tubular epithelial (HK-2) cells, downregulation of heat shock protein A12A (HSPA12A) reduced extracellular lactate post-H/R, while HSPA12A overexpression increased glycolysis and cell proliferation via HIF-1α (Min et al., 2024).

Proximal tubule-specific HIF-1α knockout by the Cre‐LoxP recombination strategy in streptozotocin injection induced diabetic mice worsened renal injury, mitochondrial fragmentation, ROS, and apoptosis; HIF-1α overexpression or HO-1 activation reversed mitochondrial damage (Jiang N. et al., 2020). During AKI, HIF-1α driven metabolic reprogramming in proximal tubules enhances glycolysus while suppressing fatty acid oxidation (FAO) through downregulation of FAO-related transcription factors (Piret and Mallipattu, 2023).

### 5.3 HIF-1α and necroptosis in acute kidney injury

Necroptosis, a regulated form of necrosis mediated by the RIPK1–RIPK3–MLKL signaling cascade, is increasingly acknowledged as a significant factor in renal tubular injury associated with AKI (Liu et al., 2022). Hypoxia and the stabilization of HIF-1α can directly enhance this pathway through distinct molecular mechanisms. A pivotal study demonstrates that under conditions of prolonged hypoxia, the activation of HIF-1α is linked to necroptosis via the oxygen-sensing machinery itself: the prolyl hydroxylase EGLN1 and its recognition partner pVHL, which typically hydroxylate and destabilize both HIF-1α and RIPK1 under normoxic conditions, are unable to perform these functions during hypoxia. This failure in prolyl hydroxylation directly facilitates RIPK1 activation and necrosome formation, independent of TNFα signaling, thereby connecting hypoxic stress with inflammatory cell death (Zhang et al., 2023). Beyond this direct post-translational regulation, HIF-1α exerts transcriptional control over microRNAs that influence cellular metabolism and survival decisions. In macrophages, HIF-1α upregulates miR-210 while downregulating miR-383, inducing a metabolic shift that depletes ATP and promotes necroptosis, a mechanism likely pertinent to the activation of renal immune cells during AKI (Karshovska et al., 2020). In renal tubular epithelial cells, the primary target in AKI—hypoxia-reoxygenation injury induces necroptosis, characterized by increased RIP1/RIP3 interaction and necrosome assembly, which can be attenuated by the RIPK1 inhibitor Necrostatin-1 (Zhu et al., 2018). Collectively, these findings elucidate how HIF-1α, acting as a central hypoxia-responsive node, can exacerbate renal damage by integrating metabolic reprogramming, direct kinase activation, and miRNA-mediated regulation to drive necroptosis and necroinflammation, highlighting novel therapeutic targets for AKI.

### 5.4 HIF-1α and the inflammatory response in acute kidney injury

HIF-1α is closely associated with the inflammatory response in AKI. In the LPS-induced AKI rat model, HIF-1α expression was upregulated, alongside increased levels of pro-inflammatory cytokines IL-6 and TNF-α levels and reduced levels of anti-inflammatory cytokine IL-10. Administration of HIF-1α inhibitors reversed these inflammatory factor changes, suggesting that HIF-1α activation may promote inflammatory responses (Diao et al., 2024). In SA-induced AKI, levels of spliced X-box binding protein 1 (XBP1) were elevated in renal tissue. Renal-specific overexpression of Xbp1s resulted in severe tubular dilation, vacuolar degeneration, increased expression of injury markers, kidney injury molecule-1 (KIM-1) and neutrophil gelatinase-associated lipocalin (NGAL), and elevated pro-inflammatory molecules such as Il6 and Tlr4. This was associated with a decline in renal function and a 50% mortality rate. Conversely, renal-specific XBP1 knockout alleviated LPS-induced renal injury. This indicated that XBP1 plays a crucial role in inflammation and injury in sepsis-associated AKI (Ferre et al., 2019), although it is not directly associated with HIF-1α, its mediated inflammatory response may interact with HIF-1α signalling.

In cisplatin-induced AKI, inhibition of the CXCL1-CXCR2 signaling axis attenuated renal injury by reducing cytokine expression and neutrophil infiltration. This axis regulates cisplatin-induced inflammatory responses through the P38 and NF-κB signaling pathways (Liu P. et al., 2020), and its changes may have potential links to HIF-1α regulation. Renal ischemia-reperfusion injury can trigger acute liver injury and systemic inflammatory responses, with significant increases in mRNA and protein levels of pro-inflammatory cytokines (MCP-1, TNF-α, IL-6) in renal tissue and the liver, alongside activation of nuclear factor κB in the liver (Shang et al., 2020), suggesting that the systemic inflammatory response during AKI may indirectly involve the HIF-1α regulatory network.

### 5.5 HIF-1α in apoptosis and autophagy in acute kidney injury

HIF-1α plays a key role in the regulation of apoptosis and autophagy in AKI. In an ischemia-reperfusion-induced AKI mouse model, the expression of HIF-1α and BNIP3 increased, and autophagy and endoplasmic reticulum autophagy (ER-phagy) were activated (Zhao et al., 2025; Fu et al., 2020; Li J. et al., 2023). However, HIF-1α knockout in HIF-1α^flox/flox^: cadherin16-cre^+^ mice significantly inhibited BNIP3, autophagy, and exacerbating renal injury (Fu et al., 2020). *In vitro* experiments further confirmed that overexpression of HIF-1α increased BNIP3, autophagy, and ER-phagy levels, while inhibiting BNIP3 reversed the protective effect of HIF-1α (Zhao et al., 2025). Additionally, inhibiting autophagy could counteract HIF-1α′s suppression of apoptosis, indicating that the HIF-1α/BNIP3 axis-mediated ER-phagy protected renal tubular cells from IR damage by activating autophagy and inhibiting apoptosis (Zhao et al., 2025).

**FIGURE 5:**
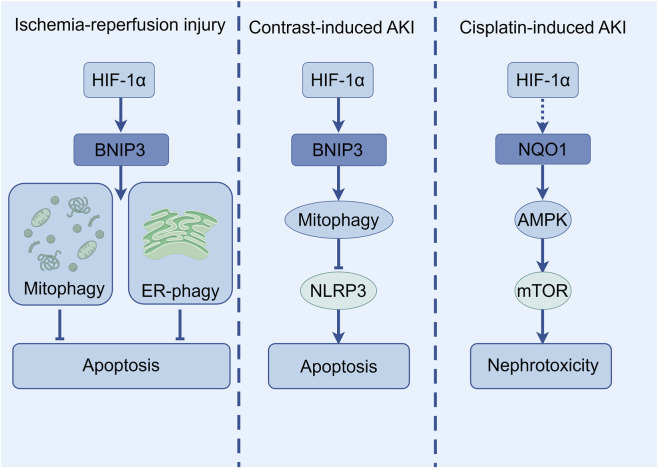
The diagram illustrates the selective autophagy pathway regulated by HIF-1α in various AKI models.

In CI-AKI, inhibition of NLRP3 inflammasome attentuated apoptosis by upregulating HIF-1α and BNIP3-mediated mitophagy. BNIP3 deficiency significantly reduced mitochondrial autophagy, exacerbating apoptosis and renal injury (Lin et al., 2021). This underscores the importance of HIF-1α-BNIP3-mitophagy axis in protecting renal tubules against apoptosis. In cisplatin-induced AKI, NAD(P)H, quinone oxidoreductase 1 (NQO1)-deficient mice exhibited more severe renal injury, accompanied by increased expression of autophagy-related proteins but impaired autophagosome maturation (as evidenced by reduced Ras-associated protein 7 expression and p62 protein accumulation), suggesting that NQO1 may regulate autophagy through the AMPK/TSC2/mTOR signaling pathway to influence cisplatin-induced nephrotoxicity (Kim et al., 2016). Although HIF-1α is not directly involved, the bidirectional regulation between autophagy and apoptosis may be potentially linked to the mechanism of HIF-1α in AKI (Figure 5).

## 6 Diagnostic techniques for HIF-1α in acute kidney injury

### 6.1 The potential of HIF-1α as a biomarker for acute kidney injury

Research on HIF-1α as a biomarker for AKI is increasingly prevalent. In an LPS-induced AKI rat model, HIF-1α protein levels in both serum and kidney tissue were significantly upregulated within 12 h, accompanied by significant alterations in metabolic and inflammatory markers (Diao et al., 2024). This suggests that dynamic changes in HIF-1α expression may reflect the onset and progression of AKI, indicating its potential value as a biomarker. In various AKI animal models and clinical studies, markers such as KIM-1 and NGAL have been confirmed to be valuable for early AKI diagnosis (Brilland et al., 2023; Allegretti et al., 2021). Although no direct comparative studies currently exist between HIF-1α and these markers, the central role of HIF-1α in AKI pathophysiology underscores its potential as a diagnostic marker deserving further exploration. In a longitudinal follow-up of 31 patients undergoing abdominal aortic aneurysm surgery, urinary fibrinogen was found to rise earlier than SCr, the standard but often delayed biomarker for AKI, in patients who developed AKI postoperatively (Hoffmann et al., 2012). Similarly, in drug-induced AKI models, the elevation of urinary vanin-1 precedes increases in SCr and other biomarkers such as urinary N-acetyl-β-D-glucosaminidase (NAG), KIM-1, and NGAL (Hosohata et al., 2012). These findings highlight the need to identify early, sensitive AKI biomarkers. The potential of HIF-1α in this context remains to be fully explored.

### 6.2 Advances in HIF-1α detection technology in acute kidney injury

Beyond conventional measures such as SCr and urine output, novel biosensors are expanding the diagnostic landscape for AKI. For instance, a Y-shaped aptamer-based surface-enhanced Raman scattering (SERS) sensor enabled highly sensitive dual-protein detection in AKI rat models and spiked human plasma samples (Li et al., 2022). Established techniques, including enzyme-linked immunosorbent assay (ELISA) and bead-based immunoassays, are routinely used to quantify urinary biomarkers such as urinary NGAL (uNGAL), urinary cystatin C (uCysC), urinary clusterin (uClusterin), urinary β_2_-microglobulin (uβ2M), and urinary KIM-1 (uKIM-1) (Mohamed et al., 2017). The commercial NephroCheck® test [urinary (TIMP-2) × (IGFBP7)] received FDA approval in 2014 (Ilaria et al., 2021), providing a model for HIF-1α-based tools.

Although studies directly applying HIF-1α measurement to the early diagnosis of AKI remain limited, existing evidence provides a strong theoretical foundation for its potential value. In an LPS-induced AKI rat model, early and significant upregulation of HIF-1α protein levels occurred synchronously with characteristic AKI pathophysiological changes, including hypoglycemia, enhanced glycolytic activity, and altered inflammatory cytokine profiles (Liu C. et al., 2020). Monitoring such dynamic changes in HIF-1α may help capture early onset of AKI. Research on other markers also supports this possibility. For example, neutrophil gelatinase-associated lipocalin has demonstrated early predictive power for AKI in various clinical scenarios such as cardiac surgery and sepsis (Snel et al., 2025), providing strong evidence for exploring key regulatory molecules like HIF-1α as early diagnostic markers. In specific AKI subtypes, such as CI-AKI, non-protein biomarkers like miRNA have also shown diagnostic potential. For example, miR-155 levels are significantly elevated in SA-AKI and correlates with the renal dysfunction severity. Its diagnostic performance, confirmed by receiver operating characteristic curve, confirms its high diagnostic value (Fan et al., 2024). These findings suggest that the diagnostic feasibility of HIF-1α could be assessed by evaluating its correlation with known early biomarkers or by characterizing its own expression patterns in the initial stages of AKI. Integrating HIF-1α with other markers (e.g., KIM-1, NGAL, or metabolites) could enhance early AKI detection models, as exemplified by the NephroCheck® approach (Ilaria et al., 2021). This integrated strategy points the way forward for combining HIF-1α with other markers to construct a more robust multivariable early warning model for AKI.

## 7 Translational prospects for HIF-1α-targeted therapy

### 7.1 HIF-1α-targeted drug development strategies

Targeting HIF-1α is emerging as a novel therapeutic strategy for AKI. In an LPS-induced AKI rat model, administration of HIF-1α inhibitors reversed hypoglycemia, normalized enhanced glycolytic activity, and rebalanced inflammatory cytokine profiles (e.g., elevated IL-6/TNF-α and decreased IL-10), indicating that inhibiting excessive HIF-1α activation is a promising therapeutic approach (Yang et al., 2025). Several compounds in early-stage clinical trials are being explored for their efficacy in alleviating kidney injury by modulating HIF-1α-related pathways (e.g., HIF-1α/KLF5, HIF-1α/NF-κB) (Lu et al., 2024). A deeper understanding of AKI pathogenesis will facilitate the development of drugs that precisely target HIF-1α (e.g., small-molecule inhibitors or activators) to correct its aberrant activity, thereby ameliorating inflammatory responses, cellular metabolic imbalances, and apoptotic processes. Progress with HIF-1α-targeting drugs in other diseases, such as the use of PHD inhibitors (e.g., roxadustat) for anemia, provides valuable insights for AKI drug development. Ultimately, rigorous clinical trials are needed to validate the efficacy and safety of these interventions in AKI patients.

### 7.2 Exploration of gene therapy regulated by HIF-1α

Gene therapy represents an emerging approach to modulate HIF-1α for AKI treatment. For instance, activation of the C-terminal transactivation domain of HIF-1α promotes AKI-to-CKD progression via the KLF5 signaling pathway, a process that can be significantly blocked by the HIF-1α inhibitor PX-478 (Li et al., 2021a). This suggests that targeted genetic intervention in HIF-1α or its downstream pathways could curb disease progression. Nanocarrier technology enables targeted delivery; for example, synthetic polydopamine-polyethyleneimine-L-serine-Klotho plasmid nanoparticles can safely and efficiently deliver the Klotho gene to damaged renal tubular epithelial cells. This approach upregulates PPARα, improving fatty acid β-oxidation, reducing lipid accumulation, and thereby preventing renal fibrosis (Li et al., 2025). Although not directly targeting HIF-1α, this metabolic restoration may synergize with HIF-1α pathways, informing the design of combinatorial gene therapies. At the microRNA level, ischemia/reperfusion injury upregulates miR-21 and HIF-1α/2α. miR-21 interacts with HIF via the PTEN/AKT/mTOR pathway, and its inhibition activates the PDCD4/NF-κB pathway, exacerbating apoptosis and inflammation (Song et al., 2018). Thus, regulating HIF-related microRNAs is a promising new direction. Optimizing delivery vehicles (e.g., viral vectors, novel nanomaterials) and strategies to enhance targeting and safety remains a critical focus for future research.

### 7.3 Current status of clinical translation of HIF-1α targeted therapy

Although clinical trials directly targeting HIF-1α for AKI are still limited, promising progress has been made. One study used ammonium-functionalized carbon nanotubes to deliver siRNA targeting *Trp53* and *Mep1b* to renal tubular cells. Prophylactic use of this fCNT/siRNA complex significantly improved survival rates with good tolerability in mouse and non-human primate models of cisplatin-induced AKI (Alidori et al., 2016). While this approach did not directly target HIF-1α, it establishes a translational foundation for developing HIF-1α-related gene silencing or activation therapies. Notably, clinical trials targeting erythropoietin showed no significant improvement in anemia, renal function recovery, or mortality in AKI patients (Aoun et al., 2022). However, their rigorous designs provide valuable experience for future HIF-1α-targeted trials. Other related strategies, such as mitochondrial-targeted drugs, do not directly involve HIF-1α but hold combinatorial potential due to the close interaction between HIF-1α signaling and mitochondrial function (Tabara et al., 2014). These studies provide a rationale for exploring combined treatment strategies (e.g., HIF stabilizers with mitochondrial protectants) and inform the design of control trials. Future work should involve larger-scale, well-designed clinical trials to verify the optimal timing of intervention (e.g., early inhibition vs. late promotion of repair), long-term safety, and the impact of HIF-1α-targeted therapies on hard renal endpoints.

### 7.4 Synthetic and natural compounds based on HIF-1α regulation

Kidney protection strategies based on HIF-1α regulation primarily include two categories: synthetic drugs and natural active compounds. HIF prolyl hydroxylase inhibitors stabilize HIF-1α, activate the HIF-1α/VEGFA/VEGFR1 signaling axis, and upregulate SOD2. In experimental models, this significantly alleviates renal fibrosis, promotes angiogenesis, and delays AKI-to-CKD progression (Zhou et al., 2018). The antidiabetic drug dapagliflozin alleviates contrast-induced AKI by inhibiting HIF-1α/HE4/NF-κB signaling (Huang X. et al., 2022). It also increases HIF1α and phosphorylated AMP-activated protein kinase (*p*-AMPK) expression in a dose-dependent manner and improves the survival rate of hypoxic tubular cells (Chang et al., 2016), suggesting that HIF-1α mediates its renal protective effects.

**FIGURE 6:**
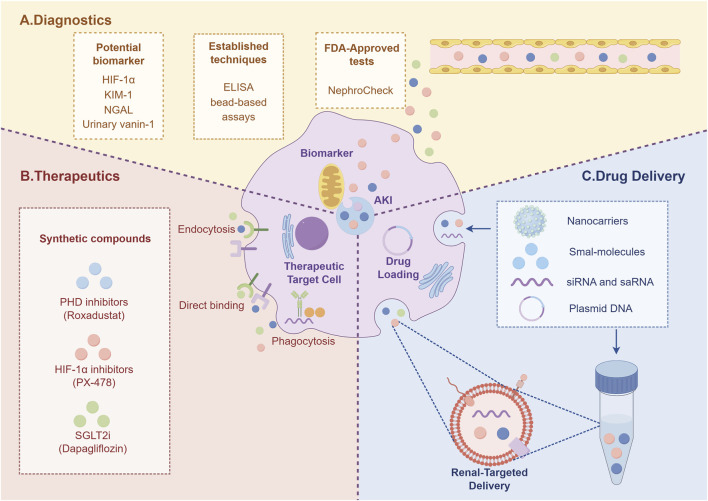
The diagram summarizes the potential of targeting HIF-1α for AKI management, encompassing novel diagnostics **(A)**, advanced therapeutics **(B)**, and delivery systems **(C)**.

Several natural products show potential in regulating HIF-1α. Quercetin alleviates contrast-induced kidney injury by inhibiting the HIF-1α/lncRNA NEAT1/HMGB1 axis (Luo et al., 2022). Resveratrol ameliorates contrast-induced AKI in diabetic nephropathy by activating the SIRT1-PGC-1α-HIF-1α pathway, mitigating hypoxia, mitochondrial dysfunction, and apoptosis (Wang et al., 2019). Ginsenosides attenuate glycerol-induced AKI and oxidative damage by upregulating HIF-1α and VEGF-A levels in the kidney-brain axis (Mao et al., 2020). Emodin promotes angiogenesis and improves renal ischemia-reperfusion injury by regulating the HIF-1α/VEGF pathway (Lu et al., 2023). The traditional Chinese medicine compound Xiao Yu Xie Zhuo Yin exerts protective effects against elderly AKI by inhibiting the TGF-β1/Smad3 and HIF1 signalling pathways (Ye et al., 2021). All-trans retinoic acid reverses cisplatin nephrotoxicity by downregulating caspase-3, IL-6, and TGF-β1 while upregulating HIF-1α, VEGF, and CD31 (Khedr et al., 2022) (Figure 6).

## 8 Future prospects for HIF-1α in acute kidney injury

### 8.1 Cutting-edge directions in HIF-1α research in acute kidney injury

Current research on HIF-1α in AKI is focused on two cutting-edge directions. First, it is crucial to thoroughly understand the mechanism of action of HIF-1α in the complex signalling network of AKI (Han et al., 2024), and its interactions and co-regulation with key molecules (such as KLF5) still need to be clarified. For example, in the specific pathway where the C-terminal transactivation domain of HIF-1α promotes the progression of AKI to chronic kidney disease, the presence of unknown intermediate steps or synergistic factors remains to be elucidated (Li et al., 2021a). Second, the cross-talk mechanisms between HIF-1α and multiple pathways such as metabolism, inflammation, and apoptosis remain unclear. Clarifying these networks will provide a theoretical foundation for developing precise treatment strategies. Breakthroughs in single-cell sequencing technology have opened up new opportunities. This technology can analyse the expression differences and functional specificity of HIF-1α in specific subpopulations such as renal tubular epithelial cells, macrophages, and fibroblasts at the single-cell level (Han et al., 2024), driving the transition of AKI treatment from broad-based interventions to cell type-targeted regulation. Based on the above understanding of the mechanism, the development of drugs targeting HIF-1α holds great promise: small-molecule compounds designed to precisely regulate its activity (such as inhibiting excessive activation) have been shown to reverse glucose metabolism disorders and inflammatory factor imbalances in LPS-induced AKI models (Li et al., 2021b).

In addition, gene editing or gene delivery therapies targeting HIF-1α are expected to become a reality in gene therapy strategies (Zhang Z. et al., 2022). Gene therapy strategies may achieve precise repair of kidney cells by targeting regulatory elements (such as the CRISPR system or HIF-1α effector genes) via nanocarriers, drawing on the successful delivery of the Klotho gene (Neyra et al., 2020). Mesenchymal stem cells and their exosomes may also promote tissue regeneration by regulating the HIF-1α network through paracrine signals (Cao et al., 2021).

### 8.2 Challenges and opportunities in HIF-1α research in acute kidney injury

HIF-1α still faces significant challenges in AKI research. Its role is highly complex: it may exert opposite functions in different disease stages (acute injury phase vs. fibrotic progression phase) and different cell types (early protection vs. sustained activation promoting fibrosis), and its interaction network with metabolic, inflammatory, and cell death pathways has not yet been fully elucidated (Jiang M. et al., 2020). For example, when targeting key nodes in HIF-1α to promote CKD progression, overcoming the challenges of its synergistic effects with multiple factors is necessary. The development of safe and effective targeted drugs also faces bottlenecks such as specificity (avoiding off-target effects), bioavailability (renal-selective delivery), and long-term safety. However, new technologies are creating breakthrough opportunities: single-cell sequencing can precisely map the cell type-specific regulatory landscape of HIF-1α (Han et al., 2024), providing a basis for designing spatiotemporally precise intervention strategies; gene editing technologies (such as CRISPR-Cas9) hold promise for targeted modification of HIF-1α or its regulatory factors; advanced nanocarriers can optimise the renal targeted delivery efficiency of therapeutic molecules (Zhang Z. et al., 2022; Neyra et al., 2020). A deeper understanding of the pathophysiology of AKI, combined with interdisciplinary technological integration, will accelerate the translation of HIF-1α research findings into transformative clinical therapies.
